# Costs of the Smoking Cessation Program in Brazil

**DOI:** 10.1590/S1518-8787.2016050006303

**Published:** 2016-10-26

**Authors:** Andréa Cristina Rosa Mendes, Cristiana Maria Toscano, Rosilene Marques de Souza Barcellos, Alvaro Luis Pereira Ribeiro, Jonas Bohn Ritzel, Valéria de Souza Cunha, Bruce Bartholow Duncan

**Affiliations:** I Departamento de Economia da Saúde, Investimentos e Desenvolvimento. Ministério da Saúde. Brasília, DF, Brasil; II Departamento de Saúde Coletiva. Instituto de Patologia Tropical e Saúde Pública. Universidade Federal de Goiás. Goiânia, GO, Brasil; III Divisão de Doenças Crônico-Degenerativas. Secretaria Municipal de Saúde de Goiânia. Goiânia, GO, Brasil; IV Instituto Nacional de Câncer José Alencar Gomes da Silva. Ministério da Saúde. Rio de Janeiro, RJ, Brasil; V Programa de Pós-Graduação em Epidemiologia. Faculdade de Medicina. Universidade Federal do Rio Grande do Sul. Porto Alegre, RS, Brasil

**Keywords:** Smoking Cessation, economics, Costs and Cost Analysis, Health Care Costs, Unified Health System, Smoking

## Abstract

**OBJECTIVE:**

To assess the costs of the Smoking Cessation Program in the Brazilian Unified Health System and estimate the cost of its full implementation in a Brazilian municipality.

**METHODS:**

The intensive behavioral therapy and treatment for smoking cessation includes consultations, cognitive-behavioral group therapy sessions, and use of medicines. The costs of care and management of the program were estimated using micro-costing methods. The full implementation of the program in the municipality of Goiania, Goias was set as its expansion to meet the demand of all smokers motivated to quit in the municipality that would seek care at Brazilian Unified Health System. We considered direct medical and non-medical costs: human resources, medicines, consumables, general expenses, transport, travels, events, and capital costs. We included costs of federal, state, and municipal levels. The perspective of the analysis was that from the Brazilian Unified Health System. Sensitivity analysis was performed by varying parameters concerning the amount of activities and resources used. Data sources included a sample of primary care health units, municipal and state secretariats of health, and the Brazilian Ministry of Health. The costs were estimated in Brazilian Real (R$) for the year of 2010.

**RESULTS:**

The cost of the program in Goiania was R$429,079, with 78.0% regarding behavioral therapy and treatment of smoking. The cost per patient was R$534, and, per quitter, R$1,435. The full implementation of the program in the municipality of Goiania would generate a cost of R$20.28 million to attend 35,323 smokers.

**CONCLUSIONS:**

The Smoking Cessation Program has good performance in terms of cost per patient that quit smoking. In view of the burden of smoking in Brazil, the treatment for smoking cessation must be considered as a priority in allocating health resources.

## RESUMO

**OBJETIVO:**

Analisar os custos do Programa de Tratamento do Tabagismo no Sistema Único de Saúde e estimar o custo de sua implementação plena em um município brasileiro.

**MÉTODOS:**

A abordagem intensiva e tratamento do tabagismo engloba consultas, sessões de terapia cognitivo-comportamental em grupo e uso de medicamentos. Os custos do atendimento e gerenciamento do programa foram estimados utilizando a metodologia do microcusteio. A implementação plena do programa no município de Goiânia, Goiás, foi definida como sua expansão para suprir a demanda de todos os fumantes motivados a parar de fumar no município que seriam atendidos pelo Sistema Único de Saúde. Foram considerados custos médicos e não médicos diretos: recursos humanos, medicamentos, material de consumo, despesas gerais, transporte, viagens, eventos e custos de capital. Foram incluídos custos dos níveis federal, estadual e municipal de gestão. A perspectiva da análise foi a do Sistema Único de Saúde. Análise de sensibilidade foi realizada variando parâmetros referentes à quantidade de atividades e aos recursos utilizados. As fontes de dados incluíram uma amostra de unidades de saúde da Atenção Primária, secretarias de saúde municipal e estadual e Ministério da Saúde. Os custos foram estimados em reais (R$) para o ano de 2010.

**RESULTADOS:**

O custo do programa em Goiânia foi de R$429.079, sendo 78,0% referentes à abordagem e tratamento do tabagismo. O custo por paciente foi de R$534 e, por paciente que deixou de fumar, de R$1.435. A implementação plena do programa no município de Goiânia geraria custo de R$20,28 milhões, para atender 35.323 fumantes.

**CONCLUSÕES:**

O Programa de Tratamento do Tabagismo tem bom desempenho em termos de custo por paciente que deixa de fumar. Tendo em vista a carga do tabagismo no Brasil, o tratamento para cessação de fumar deve ser considerado prioritário ao se programar a alocação de recursos de saúde.

## INTRODUCTION

Smoking is an epidemic disease arising from nicotine addiction and is the leading cause of preventable deaths in the world, estimated in 6 million annually^a^. It is estimated that it will be responsible for more 175 million deaths between 2005 and 2030^b^.

The economic burden of smoking to society is also significant. The annual expenses with treatment of tobacco-related diseases and the loss of productivity resulting from the morbidity and mortality of smoking have generated an economic burden of U$193 billion in the United States between 2000-2004^1^ and of €21 billion in Germany, in 2003^13^.

In Brazil, smoking resulted in 130,000 deaths in 2008, 13.0% of total deaths, already surpassing international estimates of 10.0% of deaths attributable to tobacco in 2015. The costs of treating tobacco-related diseases amounted to R$21 billion in 2011, considering the public and supplemental health systems^11^ ^,^ ^c^.

Since the 1980s, the Brazilian Ministry of Health has been adopting various actions for smoking control, which culminated in the creation of the National Smoking Control Program, coordinated by the National Cancer Institute (INCA) since the early 1990s^d^, and today part of the National Smoking Control Policy, which has an intersectoral nature. The program encompasses from comprehensive actions, as promoting smoke-free environments, to more focused ones, as promotion and support for smoking cessation. Focusing on the latter, the Smoking Cessation Program was implemented in the Brazilian Unified Health System (SUS) in 2002, to increase the access of smokers to effective methods for smoking cessation, and adopting the methods recognized as first line treatment^e^, according to recommendations of the Consensus for Approach and Treatment of Smokers^10^. In Goias, the municipality of Goiania was the first to deploy the program, figuring among the 88 municipalities with smoking treatment in Brazil in 2005^d^.

In the treatment model adopted by Brazil, after initial assessment appointment, smokers receive intensive cognitive-behavioral therapy, which combines cognitive interventions with training of behavioral skills^10^. The therapy is performed in a group from 10 to 15 smokers or individually, being composed of four structured initial sessions of 90 minutes, preferably weekly, followed by 12 sessions, until the completion of one year of treatment. Pharmacotherapy, when indicated, is made by nicotine replacement therapy (NRT), with the use of transdermal nicotine patches (21, 14, and 7 mg), nicotine gum or lozenge (2 and 4 mg), and the antidepressant bupropion hydrochloride (150 mg), used individually or in combination. The program also includes brief counseling, conducted during the routine consultations, lasting from three to five minutes^f^.

The program is managed articulately by the federal, state, and municipal levels of SUS management. The trainings, technical support to activities, and distribution of used inputs are done “cascading”, from the federal to the municipal level. Educational materials and medicines are supplied by the Ministry of Health, and may be supplemented by the states and municipalities. The information is collected and consolidated from the local to the central level.

The effectiveness of brief counseling, intensive group therapy, NRT, and bupropion was evidenced by several meta-analyses^3^ ^,^ ^23^ ^,^ ^26^. The combination of behavioral therapy with the use of medicines is recommended, showing slightly better results when the behavioral therapy is more intensive^24^ ^,^ ^25^. In Brazil, cessation rates were reported between 23.5% and 50.8%, after at least six months from the beginning of treatment^14^ ^,^ ^20^ ^,^ ^21^.

In view of the scarcity of resources for health, as well as of effectiveness, we must also consider the costs of health programs. These are still an essential part of health economic evaluations that compare treatment alternatives, producing information on the costs and benefits of them, thus orienting the decision-making^7^.

Studies conducted in the United States indicated costs of smoking treatment programs per participant of U$67 (brief telephone counseling without NRT), U$268 (intensive counseling associated with NRT)^8^, and U$183 (telephone counseling, pharmacotherapy, and promotion and advertising)^18^. In Brazil, there are no studies so far that assess the cost of the program implemented on SUS.

The cost estimate of the Smoking Cessation Program in Brazil is, therefore, an important subsidy to support SUS managers in planning actions and maximizing the use of resources, as well as in decision-making regarding their prioritization, guiding the efficient allocation of resources.

The aim of this study was to estimate the costs of the program in a Brazilian municipality, including the treatment of the smoker and program management, considering the different levels of management involved. We also estimated costs if the service was expanded to fully meet the demand of the municipality’s smokers motivated to quit.

## METHODS

Cost study of the Smoking Cessation Program, implemented in primary health care. The unit of analysis was the municipality of Goiania, adopting the year of 2010 as time frame, both for measurement of data and valuation of inputs. We considered the perspective of SUS, funder of the program in the public health sector in Brazil, including direct medical and non-medical costs and management costs. Indirect costs were not considered.

The activities were identified and grouped into components of the two program axes: behavioral therapy and treatment of smoking and management. The behavioral therapy and treatment axis comprises the following components: (i) consultations (medical and nursing), including initial clinical evaluation and return visits; (ii) intensive cognitive-behavioral therapy sessions, including their preparation and execution; and (iii) phamacotherapy. In Goiania, the group sessions are conducted over six months. The brief counseling was not considered. The management axis includes two components: (i) technical support and (ii) training.

The costs of behavioral therapy and treatment of smoking were estimated considering a convenience sample of health facilities and extrapolated to the municipality as a whole, also using data available to all groups and patients attended. The sample was selected considering the following criteria: (i) systematization and availability of information; (ii) time of implementation of the program; (iii) quantity of groups; (iv) type of health facility; and (v) location. Six facilities were selected, considering different types and locations, with the largest number of groups, deployment time, and better quality of information. The program management costs were was estimated considering the activities carried out by municipal, state, and federal coordinations of the program.

The activities of the two axes of the program were estimated by micro-costing, measuring the amount used of each resource and assigning it the corresponding unit value. The resources used were identified as cost items and categorized as medical and non-medical costs, considering recurrent and capital costs. The cost structure, according to the components and axes of the program, is presented in Table 1. We included the costs incurred by the three levels of SUS management.

**Table 1:** Structure of costs considered for the costs estimation, according to the axes of the Smoking Cessation Program. Goiania, GO, Midwestern Brazil, 2010.

Group of cost items	Cost items
Behavioral therapy and treatment of smoking^a^	
Medical costs	
Healthcare human resources	Human resources directly involved in the consultations, sessions, and dispensation of medicines
Nicotine replacement therapy	Nicotine patch, lozenge, and gum
Antidepressant	Bupropion hydrochloride
Medical equipment and office furniture	Anthropometric scale, pressure equipment, stethoscope, stretchers, and others
Non-medical costs	
Administrative human resources	Administrative services and support activities (hygiene and cleaning)
Consumables	Office, pantry, and kitchen supplies and program manuals
General expenses	Rent, water, electricity, telephone, internet, gas
Office equipment and furniture	General furniture, computers and peripherals, air conditioning, among others
Management^b^	
Non-medical costs	
Human resources	Exclusive and partly involved in the program human resources
Consumables	Office, pantry, and kitchen supplies
General expenses	Water, electricity, telephone, internet, surveillance, cleaning
Transport	Taxes, fuel, and maintenance
Travels	Tickets and daily rates
Events	Rental of space and equipment, feeding, among others
Equipment and vehicles	Equipment, furniture, and vehicles

^a^ Includes consultations, cognitive-behavioral therapy sessions, and pharmacotherapy.

^b^ Includes technical support and training.

Shared costs with other health programs and actions were assigned to the Smoking Cessation Program using as an indicator of the attributable fraction the proportion of professionals’ time dedicated to the program in relation to the total time of the goal activities of the health unit or, in the case of management, of the administrative sector where the coordination of the program is.

In the sampled health facilities, the costing of the behavioral therapy and treatment was made by therapeutic group, according to the specific care configuration adopted in the facility regarding the frequency and duration of activities and professionals involved. The data were obtained in interviews with the coordinators of the groups. The professionals’ time dedicated to the cognitive-behavioral therapy sessions was obtained in individual interviews, considering the average time for the cost estimate. The time of the consultations and of the dispensation of medicines was arbitrated considering the literature^22^ ^,^ ^g^. The remuneration of health professionals was obtained in the human resources information systems of the Municipal Secretariat of Health of Goiania (SMS) and of the State Secretariat of Health of Goias (SES). The source of information for prices of medicines was the Bank of Health Prices (BPS)^h^, from the Ministry of Health. Other data were obtained from management information systems and administrative records of SMS, SES, and Ministry of Health.

To estimate the cost of the behavioral therapy and treatment in the municipality as a whole, we considered that all smokers attended in 2010 (n = 803) underwent a common configuration of care. The parameters used in the base scenario of costs estimate of each component of behavioral and treatment and the respective data source are presented in Table 2.

**Table 2:** Parameters used in the costs estimation, according to component of the behavioral therapy and treatment of smoking and cost type. Goiania, GO, Midwestern Brazil, 2010.

Parameter	Data source
Consultations	
Medical costs	
Number of consultations per patient	Sample of health facilities
Duration of consultations^a^	Arbitrated according to parameters of the Ordinance 1,101/GM, from June 12, 2002.
Remuneration of human resources^b^	Human Resources Information System of the municipality^c^
Clinic equipment cost per patient	Sample of health units
Number of patients seen	Spreadsheets standardized by the National Cancer Institute (INCA) and by the municipal coordination for follow-up of the program^d^
Non-medical costs	
Percentage of non-medical costs of the consultations	Sample of health facilities
Cognitive-behavioral therapy sessions	
Medical costs	
Number of sessions per group	Sample of health facilities
Number of human resources per session^b^	Municipal Coordination of the program^d^
Duration of the execution of each session^a^	Sample of health faciliites
Percentage of time of preparation in relation to the execution of the sessions	Sample of health facilities
Remuneration of human resources^b^	Human Resources Information System of the municipality^c,e^
Number of therapeutic groups conducted	Spreadsheets standardized by the National Cancer Institute (INCA) and by the municipal coordination for follow-up of the program^d^
Non-medical costs	
Percentage of non-medical costs of the sessions	Sample of health facilities
Medicine treatment	
Medical costs	
Average number of dispensations per patient	System of Dispensation of Medicines (SISDM)^f^
Duration of dispensation^a^	Arbitrated, considering available evidence^20^
Remuneration of human resources	Human Resources Information System of the municipality^c^
Average cost with nicotine replacement therapy and antidepressants per patient	SISDM for quantity of users and medicines^f,g^ and Bank of Health Prices for price
Number of patients that received medicines	SISDM^f^

^a^ Time measured in minutes.

^b^ Includes high-level and middle-level professionals.

^c^ Data available for 82.9% of the units that conducted groups.

^d^ Data available for all groups conducted.

^e^ Considering the occupational categories defined by the Ordinance SAS/MS 442, from August 13, 2004, and all the categories of technical level of the assistance activities.

^f^ Data available for all patients who used medicines.

^g^ Including the losses reported in the spreadsheets standardized by INCA.

The cost with human resources was obtained by multiplying the frequency of the activity by the number of professionals involved, by the time of duration, and by the average remuneration of professionals, plus employers’ charges. The remaining medical costs were added to the value obtained (cost with clinic equipment for consultations and cost with NRT and antidepressants for medicine treatment). Non-medical costs were estimated based on the percentage they represented in relation to medical costs in the sampled health facilities. The sum of medical and non-medical costs was multiplied by the number of patients seen, for consultations and pharmacotherapy, and by the number of groups (n = 44), for sessions. In the consultations, patients receiving or not medicines were stratified, considering that patients present at the fourth session of cognitive-behavioral therapy made return visits. The sum of the total cost with consultations, sessions of behavioral therapy, and pharmacotherapy resulted in the total cost of the therapy and treatment of smoking.

Management costs were estimated for each realm of management. The data were obtained with the program coordinators, in administrative records and management information systems of the three realms. The fraction of federal and state cost attributable to the municipality of Goiania was obtained based on the number of cities managed by these coordinations. The total of management assigned to the municipality was divided between the groups conducted.

The Figure shows the analysis of the cost estimate, considering the distribution of all patients seen by the components of the intensive behavioral therapy and treatment of smoking (A) and the final cost estimate of the Smoking Cessation Program, including management (B).

**Figure:**
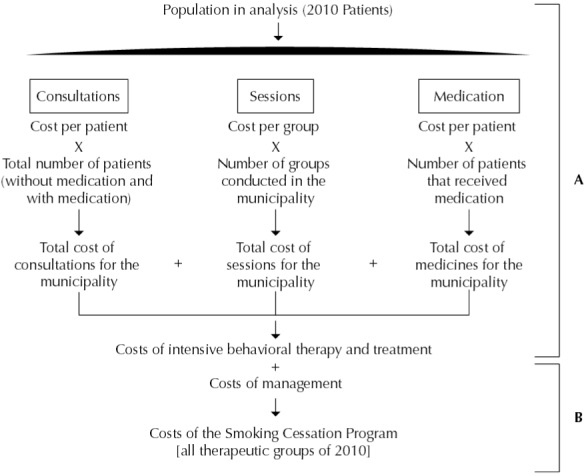
Schematic representation of the cost estimation analysis of the Smoking Cessation Program. Goiania, GO, Midwestern Brazil, 2010.

The results are presented as total cost of the program, per group, stratified by cost item, component, and funding source. Cost per patient was estimated by dividing the cost of the program among all patients attended at the first session of cognitive-behavioral therapy. The cost per patient that quit smoking corresponds to the total cost divided by the patients who were not smoking at the end of the treatment.

The expansion of the program to meet the demand of all smokers motivated to quit smoking in the municipality was set as full implementation. For the estimation of the number of smokers to be attended, we considered the prevalence of smokers in the population with 15 years or more of age (17.0%)^i^ ^,^ ^j^, the percentage of those trying to quit smoking (41.8%)^i^ ^,^ ^j^, discounting the natural cessation rate, i.e., those who would stop smoking without treatment (2.5%)^5^, and individuals previously attended in 2010 (803). We considered the smokers who were potential users of SUS for the treatment (50.0%)^i^.

The smokers were distributed by the three components of the behavioral therapy and treatment, according to common care configuration of the municipality, considering maximum efficiency: all primary care facilities conducting two groups of 15 smokers per year, with provision of medicines to 84.0% of them, according to the degree of dependence to nicotine of the 2010 patients, measured by Fagerström test. The cost of management was estimated by multiplying the annual cost of 2010 by the number of years required for all smokers to access treatment.

A sensitivity analysis was performed to assess the impact of the variation of parameters on the results of the behavioral therapy and treatment costs. Many parameters not known or with different values on different sources of information were analyzed. In addition to the base scenario, three new scenarios were created: (i) SMS-Goiania Guidance – considering recommendations or administrative records of the municipal coordination of the program; (ii-iii) Minimum and Maximum, considering respectively minimum and maximum values found in the sampled units and data sources (Table 3).

**Table 3:** Various parameters in the sensitivity analysis to estimate the costs of the Smoking Cessation Program in the different considered scenarios. Goiania, GO, Midwestern Brazil, 2010.

Parameter	Base scenario	Guidelines SMS-Goiania scenario	Minimum scenario	Maximum scenario
Number of consultations of clinical evaluation per patient				
Patient without medication and with medication				
Medical consultations	1.0	1.0	1.0	1.0
Nursing consultations	0.6	1.0	0	1.0
Number of return visits per patient				
Patient without medication				
Medical consultations	1.7	1.0	0	5.0
Nursing consultations	1.4	1.0	0	5.0
Patient with medication				
Medical consultations	5.9	3.0	4.0	8.0
Nursing consultations	2.3	3.0	0	8.0
Number of patients in the initial clinical evaluation				
Patient without medication	167	145	167	145
Patient with medication	463	485	463	485
Number of patients followed up in the return visits				
Patient without medication	87	65	87	65
Patient with medication	463	485	463	485
Medical equipment and office furniture costs per patient (in R$)				
Patient without medication	0.011	0.011	0.002	0.037
Patient with medication	0.016	0.016	0.005	0.045
% Non-medical costs of the consultations				
Patient without medication	15.0%	15.0%	7.0%	32.0%
Patient with medication	19.0%	19.0%	7.0%	32.0%
Number of sessions	16	18	12	20
Average number of professionals involved in the implementation of the sessions				
High-level professionals	2.3	2.3	2.3	2.3
Middle-level professionals	0.4	1.0	0	1.0
Average duration of professionals participation in the execution of the sessions (minutes)				
High-level professionals	89	89	69	117
Middle-level professionals	40	40	25	105
Percentage of preparation time in relation to the execution of the sessions				
High-level professionals	54.0%	54.0%	35.0%	74.0%
Middle-level professionals	18.0%	18.0%	0%	47.0%
% Non-medical costs of the sessions	25.0%	25.0%	12,0%	36.0%
Average cost with medicines and prescription per patient (in R$)	174	194	174	194
Number of patients with medication	463	485	463	485

SMS: Municipal Secretariat of Health

The research project was approved by the Research Ethics Committee of the Hospital das Clínicas of the Universidade Federal de Goias (Protocol 155/2011).

## RESULTS

In the sampled health facilities, the cost of the behavioral therapy and treatment per therapeutic group, composed of eight to 36 smokers, ranged from R$2,937 to R$9,864. From this total, medical costs accounted for, in average, 85.8% and human resources costs, 73.1% (ranging from 62.3% to 90.2%). Including management costs, the total cost of the Smoking Cessation Program per group ranged between R$5,130 and R$12,057. The greater participation in the funding of the program came from the municipality, responsible for 78.2% of the total cost, followed by the federal (13.6%) and state level (8.4%). The largest federal participation was related to medication, and the Ministry of Health was responsible for 67.7% of the total cost with NRT and bupropion.

In the municipality as a whole, 44 groups of six to 55 smokers were conducted, generating a total program cost of R$429,079 in the base scenario, with 78.0% regarding the behavioral therapy and treatment for smoking cessation. The component of greater participation in the costs of the behavioral therapy and treatment were of cognitive-behavioral therapy sessions (54.0%), followed by pharmacotherapy (24.3%), and consultations (21.7%). The average cost per patient was R$ 534, and, per quitter, R$1,435. The average cost of pharmacotherapy per patient who used NRT or bupropion was R$174. In the sensitivity analysis, the results varied in the minimum and maximum scenarios, but not in the SMS-Goiania Guidance scenario (Table 4).

**Table 4:** Total cost of the Smoking Cessation Program in the municipality, according to the scenarios adopted. Goiania, GO, Midwestern Brazil, 2010.

Program axes and components	Base scenario of extrapolation	Sensitivity analysis		

		Guidelines SMS-Goiania scenario	Minimum scenario	Maximum scenario
Behavioral therapy and treatment				
Consultations	72,143	49,777	48,367	108,607
Behavioral therapy sessions	179,735	206,691	80,799	390,874
Pharmacotherapy	80,686	93,978	80,686	93,978
Subtotal	332,564	350,447	209,852	593,459
Management				
Subtotal	96,515	96,515	96,515	96,515
Total cost	429,079	446,962	306,367	689,975
Patients seen	803	803	803	803
Cost per patient seen	534	557	382	859
Patients without smoking*	299	299	299	299

Cost per quitter	1,435	1,495	1,025	2,308

SMS: Municipal Secretariat of Health

*Refers to the abstinence at the end of the therapeutic group of smoking behavioral therapy and treatment.

The full implementation of the program would require R$20.28 million, benefiting 35,323 smokers motivated to quit smoking, out of a total of 175,331 smokers, representing R$574 per patient. From this total, the behavioral therapy and treatment cost would be R$19.05 million, and the management cost, R$1.24 million. In the sensitivity analysis, the total cost ranged between R$13.61 and R$34.35 million

## DISCUSSION

This study contributes to the knowledge of the costs of the Smoking Cessation Program in Brazil, estimating the costs not only of treatment, but also of the program management in the three management levels, with cost per patient that quit smoking close to the smaller ones referred to in the international literature.

The comparison of the results with the literature is difficult, given the methodological variations concerning the considered perspective of analysis, assumptions, costing method, and cost items^19^. There is also diversity in the format of programs evaluated in different countries, complicating the external validity of the results from different studies. Estimates of cost per quitter in programs with behavioral therapy generally less intensive than in Brazil, not including management, ranged between U$1,353 and U$3,596^2^ ^,^ ^6^ ^,^ ^27^. In Minnesota, USA, the cost per participant and per quitter was U$352 and U$1,934, respectively, including NRT, bupropion, minimal approach, and telephone counseling sessions^2^. In Switzerland, these costs were of U$1,182 and U$1,353, respectively, including NRT and nine counseling sessions^27^. Cost estimate for implementation of the recommendations proposed in guideline of the USA^k^, in the format that most closely matches the Brazilian program (intensive counseling with seven sessions in group and NRT), was of 2,310 and U$3,596 per quitter, considering nicotine patch and gum, respectively^6^.

The proportion of costs with pharmacotherapy in Goiania (24.3%) was not high, resembling the observed in the program evaluated in Switzerland (22.8%)^27^. The greater participation of the group sessions, responsible for 54.0% of the intervention cost, associated to the proportion of costs with human resources (73.1%), observed among the sampled facilities in this study and similar to that found in the Family Health Program of Porto Alegre in 2002 (65.5%)^4^, indicates the importance of maximizing the use of professionals’ time. Such evidence strengthen the relevance of appropriate planning of the actions carried out in the municipality, responsible for 78.0% of the total cost of the program.

The cost with medicines per patient, including NRT and bupropion, which ranged from R$174 to R$194 in the different scenarios, differs from the findings of another national study^l^: R$329 and R$501 per patient for bupropion and nicotine patches, respectively. Araujo^l^ considered the complete treatment dosage, based on guidelines (and not on the actual consumption of patients), and prices applied in the Rio de Janeiro market, up to three times higher than the prices paid by the Ministry of Health. This explains the difference in the results. The centralized purchase strategy of medication by the Ministry of Health results in lower prices for medicines, contributing to lower costs of the program.

This study has strengths and limitations. The costing methodology used allowed us to identify resources with a high degree of detail, giving precision to the results, in a comprehensive approach, which also considered the program management. Some limitations arise from the difficulty of estimating the cost and the portion attributable to the program of activities, such as the dissemination and the process of buying medicines. At the federal level, we did not include transportation costs, general expenses, and capital costs. However, as these costs were not influential in the cost of the municipal and state management, we believe that they would not significantly increase the costs at the federal level. Another limitation refers to information about smoking cessation, obtained based on the patients’ self-report, without biochemistry confirmation of abstinence.

When estimating the costs of full implementation, we have incurred in uncertainties on various parameters. The number of smokers to be treated may be underestimated, because we considered only the most advanced stage of motivation for treatment, while the previous stage has been mentioned as a predictor of success^m^. It may also have been overestimated, because the number of smokers who would not need treatment to quit smoking was obtained by the natural cessation rate of the international literature, which can be higher among Brazilian smokers, given the significant decrease in the smoking prevalence experienced by the Country in recent years (35.0% between 1989 and 2003)^12^. We considered that the structure for municipal management available in 2010, higher than in previous years of implementation of the program, would be able to absorb the increased demand for treatment.

Despite the cost variation described, interventions for smoking cessation have been considered highly cost-effective^9^ ^,^ ^19^. These cost-effectiveness analyses are not transposable to Brazil. However, having in mind that, in general, they consider smoking cessation rates lower than those suggested in national studies, it is possible that the cost-effectiveness of treatment for smoking cessation in the Country is even more favorable.

In Brazil, 82.0% of lung cancer cases, one of the main tobacco-related diseases, are attributable to smoking. Their treatment in specialized public hospital was estimated in R$28,901 in 2006 for patients with a history of smoking, cost significantly higher than the cost per patient that quit smoking in the Smoking Cessation Program^15^ ^,^ ^c^. Another strategy of cancer prevention, the screening of breast cancer in women, considered a priority in recent years, had cumulative cost estimated at R$1,008 for the usual care in SUS, near the cost per patient that quit smoking^17^. The value transferred by the Ministry of Health to the municipality of Goiania in 2010 for conduction of mammograms for screening, R$1.34 million^n^, excels in three times the total cost of the Smoking Cessation Program. Although the value of transfers does not represent the cost of the intervention, but the federal contribution of resources for their realization (according to unit value set by the Ministry for compensation of the procedure), its magnitude shows the effort made for prevention of breast cancer. Considering that breast cancer resulted in 12,705 deaths in women in 2010^o^, it is important to note the potential benefit of treatment of smoking, a risk factor for chronic diseases of great world impact and responsible for 130,000 annual deaths in the Country^c^ ^,^ ^p^.

Corroborating the evidence available in the international literature, this study suggests that the Smoking Cessation Program, implemented on SUS about a decade ago, has good performance regarding the cost per patient that quit smoking in Goiania, a municipality that follows the treatment model recommended nationally. This cost can be even smaller with increasing access and demand management, diluting the costs of program management and promoting greater use of the cognitive-behavioral therapy sessions.

In view of the major impact of smoking in the morbidity and mortality of the Brazilian population, the high cost of treating tobacco-related diseases, and the favorable cost-effectiveness of interventions for treatment of smoking, the Smoking Cessation Program must be considered a priority when planning the allocation of health resources. The evidence that the total funding of treatment, i.e., without additional cost to the patient, results in higher rates of abstinence reinforces such recommendation^16^.

We suggest the conduction of studies to assess the indication of treatment of smoking for SUS users in clinical practice, assisting in the development of strategies for referencing patients and increasing the scope of the program. Also, studies about the cost-effectiveness of the program are needed to provide more subsidies for the decision-making of managers as to their prioritization and encouragement.

## Footnotes

a World Health Organization. WHO report on the global tobacco epidemic 2013: enforcing bans on tobacco advertising, promotion and sponsorship. Geneva; 2013 [cited 2014 Jun 25]. Available from: http://www.who.int/tobacco/global_report/2013/en/

b World Health Organization. WHO report on the global tobbaco epidemic, 2008: the MPOWER package. Geneva; 2008 [cited 2012 Nov 12]. Available from: http://www.who.int/tobacco/mpower/2008/en/

c Pinto MT, Pichon-Riviere A, Biz A, Schluckbier L, Araújo A. Carga das doenças tabaco-relacionadas para o Brasil: relatório final. 2012 [cited 2012 Nov 13]. Available from: http://actbr.org.br/uploads/conteudo/721_Relatorio_Carga_do_tabagismo_Brasil.pdf

d Carvalho CRS. O Instituto Nacional do Câncer e o controle do tabagismo: uma análise da gestão federal do tratamento do tabagismo no SUS [dissertation]. Rio de Janeiro (RJ): Fiocruz, Escola Nacional de Saúde Pública Sérgio Arouca; 2009.

e Ministério da Saúde, Instituto Nacional de Câncer. Programa Nacional de Controle de Tabagismo e Outros Fatores de Risco de Câncer. 2 ed. Rio de Janeiro; 2003. Available from: http://www2.inca.gov.br/wps/wcm/connect/3cd1b40047ea8d588830cd9ba9e4feaf/programa-nacional-de-controle-do-tabagismo-e-outros-fatores-de-risco-de-cancer.pdf?MOD=AJPERES&CACHEID=3cd1b40047ea8d588830cd9ba9e4feaf

f Ministério da Saúde, Secretaria de Atenção à Saúde. Portaria nº 442, de 13 de agosto de 2004. Aprova o Plano de Implantação da Abordagem e Tratamento do Tabagismo na Rede SUS, na forma do Anexo I, e o Protocolo Clínico e Diretrizes Terapêuticas – Dependência à Nicotina, na forma do Anexo II desta Portaria. *Diario Oficial Uniao*. 17 ago 2004; Seção1:62. Available from: ftp://ftp.cve.saude.sp.gov.br/doc_tec/cronicas/PortSAS442_ago04.pdf

g Ministério da Saúde. Portaria nº 1.101/GM, de 12 de junho de 2002. Estabelece os parâmetros assistenciais no âmbito do Sistema Único de Saúde. *Diario Oficial Uniao*. 13 jun 2002; Seção 1:36. Available from: http://www1.saude.ba.gov.br/regulasaude/2009/PN%20PORTARIAS%202009/nvos%20pdfs%202009/PT%20GM%201101%2012.06.2002.pdf

h Ministério da Saúde. Secretaria Executiva. Departamento de Economia da Saúde, Investimentos e Desenvolvimento. Banco de preços em saúde. Brasília (DF): Ministério da Saúde; 2012 [cited 2012 Feb 25]. Available from: http://aplicacao.saude.gov.br/bps/login.jsf

i Ministério da Saúde, Instituto Nacional de Câncer; Organização Pan-Americana da Saúde. Pesquisa Especial de Tabagismo – PETab: relatório Brasil. Rio de Janeiro (RJ): INCA; 2011. Available from: http://bvsms.saude.gov.br/bvs/publicacoes/pesquisa_especial_tabagismo_petab.pdf

j This research’s obtained parameters refer to the state of Goias and were used as proxy for the municipality of Goiania.

k Fiore MC, Bailey WC, Cohen SJ, Dorfman SF, Goldstein MG, Gritzer, et al. Smoking cessation: clinical practice guideline n 18. Rockville, MD: US Department of Health and Human Services; Agency for Health Care Policy and Research; 1996 (AHCPR Publication n 96-0692).

l Araújo AJ. Custo-efetividade de intervenções de controle de tabaco no Brasil [these]. Rio de Janeiro (RJ): Universidade Federal do Rio de Janeiro; 2008.

m Santos JDP. Avaliação da efetividade do programa de tratamento do tabagismo no Sistema Único de Saúde [dissertation]. Porto Alegre (RS): Universidade Federal do Rio Grande do Sul, Faculdade de Medicina; 2011.

n Ministério da Saúde, Departamento de Informática do Sistema Único de Saúde - DATASUS. Informações de Saúde (TABNET): assistência à saúde [cited 2014 Oct 25]. Available from: http://www2.datasus.gov.br/DATASUS/index.php?area=0202

o Ministério da Saúde, Departamento de Informática do Sistema Único de Saúde - DATASUS. Informações de Saúde (TABNET): estatísticas vitais [cited 2014 Oct 25]. Available from: www2.datasus.gov.br/DATASUS/index.php?area=0205

p World Health Organization. Global health risks: mortality and burden of disease attributable to selected major risks. Geneva; 2009 [cited 2014 Oct 7]. Available from: http://www.who.int/healthinfo/global_burden_disease/GlobalHealthRisks_report_full.pdf
